# Clinical Spectrum, Etiology, and Outcome of Neurological Disorders in the Rural Hospital of Mosango, the Democratic Republic of Congo

**DOI:** 10.4269/ajtmh.17-0375

**Published:** 2017-08-14

**Authors:** Deby Mukendi, Jean-Roger Lilo Kalo, Alain Mpanya, Luigi Minikulu, Tharcisse Kayembe, Pascal Lutumba, Barbara Barbé, Philippe Gillet, Jan Jacobs, Harry Van Loen, Cédric P. Yansouni, François Chappuis, Raffaella Ravinetto, Kristien Verdonck, Marleen Boelaert, Andrea S. Winkler, Emmanuel Bottieau

**Affiliations:** 1 Institut National de Recherche Biomédicale, Kinshasa, DR Congo;; 2 Université de Kinshasa, Kinshasa, DR Congo;; 3 Department of Clinical Sciences, Institute of Tropical Medicine, Antwerp, Belgium;; 4 Department of Microbiology and Immunology, KU Leuven, Leuven, Belgium;; 5 JD MacLean Centre for Tropical Diseases, McGill University Health Centre, Montreal, Canada;; 6 Division of Tropical and Humanitarian Medicine, Geneva University Hospitals and University of Geneva, Geneva, Switzerland;; 7 Department of Public Health, Institute of Tropical Medicine, Antwerp, Belgium;; 8 Department of Neurology, Technical University of Munich, Munich, Germany;; 9 Centre for Global Health, University of Oslo, Oslo, Norway

## Abstract

There is little published information on the epidemiology of neurological disorders in rural Central Africa, although the burden is considered to be substantial. This study aimed to investigate the pattern, etiology, and outcome of neurological disorders in children > 5 years and adults admitted to the rural hospital of Mosango, province of Kwilu, Democratic Republic of Congo, with a focus on severe and treatable infections of the central nervous system (CNS). From September 2012 to January 2015, 351 consecutive patients hospitalized for recent and/or ongoing neurological disorder were prospectively evaluated by a neurologist, subjected to a set of reference diagnostic tests in blood or cerebrospinal fluid, and followed-up for 3–6 months after discharge. No neuroimaging was available. Severe headache (199, 56.7%), gait/walking disorders (97, 27.6%), epileptic seizure (87, 24.8%), and focal neurological deficit (86, 24.5%) were the predominant presentations, often in combination. Infections of the CNS were documented in 63 (17.9%) patients and mainly included bacterial meningitis and unspecified meningoencephalitis (33, 9.4%), second-stage human African trypanosomiasis (10, 2.8%), and human immunodeficiency virus (HIV)-related neurological disorders (10, 2.8%). Other focal/systemic infections with neurological manifestations were diagnosed in an additional 60 (17.1%) cases. The leading noncommunicable conditions were epilepsy (61, 17.3%), psychiatric disorders (56, 16.0%), and cerebrovascular accident (23, 6.6%). Overall fatality rate was 8.2% (29/351), but up to 23.8% for CNS infections. Sequelae were observed in 76 (21.6%) patients. Clinical presentations and etiologies of neurological disorders were very diverse in this rural Central African setting and caused considerable mortality and morbidity.

## INTRODUCTION

Neurological disorders may include a wide range of infectious and noncommunicable diseases. In 2010, cerebrovascular disease, epilepsy/migraine, neurological degenerative conditions, and infections of the central nervous system (CNS) accounted altogether for at least 250 million disability-adjusted life years (DALYs), corresponding to 5–10% of global DALYs.^1^ It is usually considered that the prevalence and impact of neurological disorders are proportionally greatest in low-income countries, with an etiological pattern skewed toward infections of the CNS.^2,3^ However, in resource-poor settings lacking diagnostic facilities and specialized expertise,^4^ the epidemiological knowledge of neurological disorders has remained limited. In sub-Saharan Africa, they accounted for 5–25% of all admissions in the hospital-based studies of the past 20 years,^5–13^ but almost all were retrospective and conducted in teaching urban hospitals. Very few studies took place in rural areas, where comprehensive information on the burden and pattern of neurological disorders remains virtually absent.^7,10^

It can be speculated that infections of the CNS such as cerebral malaria, bacterial meningitis, human African trypanosomiasis (HAT), or human immunodeficiency virus (HIV)-related opportunistic infections represent an important proportion of the neurological case load in rural Africa.^14^ Because most of them are severe and treatable, they should be given the highest priority in the diagnostic approach. Indeed, if unrecognized and left untreated, death or severe sequelae usually occur, whereas timely specific treatment may substantially improve the outcome even in low-resource settings. Unfortunately, CNS infections often present, at least in their early stages, with nonspecific manifestations that may overlap with a myriad of other etiologies, further contributing to disastrous diagnostic delays for the patient^15^ and to a distressing feeling for the first-line caregivers.^16^

From 2011 to 2016, the project “Better Diagnosis for Neglected Infectious Diseases” (NIDIAG, www.nidiag.org) was conducted in four African and three Asian countries.^17^ Its main objective was to improve the field diagnosis of major neglected tropical/infectious diseases that are part of the differential diagnoses of three challenging clinical syndromes in low-resource settings (neurological disorders, persistent fever, and chronic abdominal pain/diarrhea). At each site, prospective clinical and diagnostic studies were carried out using an extensive set of reference tests targeting the priority (e.g., severe and treatable) conditions. This article reports on the study of neurological disorders which took place in the general referral hospital of Mosango, situated in the rural province of Kwilu (formerly a part of Bandundu province), Democratic Republic of Congo (DRC). The study objectives were to investigate the clinical spectrum, etiologies, and outcome of neurological disorders observed in this setting, as well as to identify the factors associated with death.

## MATERIALS AND METHODS

### Study design.

This study was hospital based and had a prospective observational design, with an outcome assessment at 3 and 6 months after discharge. We present our observations in accordance with the “Strengthening the Reporting of Observational Studies in Epidemiology” guideline and its recent adaptation to the field of neuroepidemiology (Standards of Reporting of Neurological Disorders).^18^

### Study setting and population.

This study was conducted at the “Hôpital Général de Référence” (HGR) of Mosango, a 350-bed rural hospital located in the province of Kwilu, in the western part of DRC, at about 400 km from Kinshasa. The hospital is run by general practitioners and provides general referral care to about 110,000 inhabitants in a catchment area of approximately 3,350 km^2^. Basic laboratory tests, conventional X-rays, and abdominal ultrasonography can be done locally, but with very limited electricity supply. There are neither imaging nor electrophysiological facilities available for advanced neurological care.

The study population consisted of patients older than 5 years presenting spontaneously, or referred from primary health care facilities, to the HGR of Mosango with neurological disorders. Inclusion criteria were purposively broad, to capture the main endemic neurological infections at an early stage. The precise study definitions and the rationale of all inclusion and exclusion criteria are reported in Supplemental Table 1. Briefly, for study eligibility, at least one of the following symptoms or signs had to be present: 1) altered state of consciousness, 2) changes in sleep pattern, 3) cognitive decline, 4) changes in personality/behavior, 5) recent epileptic seizure (within less than 2 weeks), 6) recent, severe and progressive headache, 7) meningism, 8) new onset cranial nerve lesion(s), 9) new onset sensory-motor focal deficits, and 10) new onset gait/walking disorders. Symptoms/signs had to be either of recent onset or of longer duration (weeks or even months) provided that they were still ongoing at study inclusion. From a clinical perspective, they were severe enough to warrant admission and further evaluation including a diagnostic lumbar puncture (in a setting where the decision cannot be supported by neuroimaging). Exclusion criteria were age of 5 years or below, neurological symptoms/signs related to an obvious recent physical or psychological trauma, chronic sequelae of a past neurological event (e.g., stroke), first seizure having occurred at the age of 5 years or below, and unwillingness or inability to comply with the study requirements (Supplemental Table 1).

### Case definitions of priority conditions and other diseases.

We established a list of priority conditions corresponding to severe and treatable infections of the CNS of proven or suspected epidemiological importance in Central Africa, and for which a robust diagnosis and specific treatment were feasible without advanced facilities.^14^ The priority conditions were 1) second-stage HAT, 2) cerebral malaria, 3) bacterial meningitis and unspecified meningoencephalitis, 4) tuberculosis of the CNS, 5) neurosyphilis, and 6) HIV-related neurological disorders. The diagnosis was considered as confirmed or probable according to stringent composite case definitions based on pathogen demonstration either at the study site or in external reference laboratories in Kinshasa, DRC, or in Antwerp, Belgium (detailed description in Supplemental Table 2).

For neurological and systemic disorders other than the priority conditions, diagnosis was first made on site by the study neurologist (DM), according to predefined field-adapted case definitions (Supplemental Tables 3 and 4), in the absence of confirmatory tests for most diseases. Whenever possible, an etiological diagnosis was proposed if a combination of presenting features was considered sufficiently specific (e.g., tetanus and cerebrovascular accident). If not, a syndromic diagnosis, either neurological or systemic, was allocated, relying on criteria that best described the case under study. All case record forms were then reviewed for consistency by the study supervisor, specialized in internal medicine (EB), and the conclusions obtained jointly by both the study neurologist and the supervisor were subsequently critically revised by another two experts blinded for the provisory diagnoses, one infectious disease specialist (CY), and one senior neurologist (ASW). The final diagnostic ascertainment was obtained by consensus. In case of coinfection, the most severe disease was kept as the final diagnosis.

### Patient management.

During the study period, all general practitioners of the hospital called the study neurologist, or his deputy (LM) if absent, for any patient presenting with neurological symptoms or signs in the emergency ward or in the outpatient clinic. This strategy was set up to maximize the capture of eligible patients. After careful assessment for eligibility, the investigator explained the study purpose and procedures to the potential participant with the help of a translator if necessary. Only patients for whom a written informed consent was obtained (from themselves or their legal representatives) were consecutively enrolled in the study. After inclusion, the study neurologist undertook the full clinical evaluation and the initial data collection. A standardized set of laboratory tests was then systematically performed at the study site by a dedicated and specifically trained laboratory technician (JLRK) according to preestablished standard operating procedures. It consisted of basic tests (blood cell count and differentiation, liver and kidney function tests, etc.), screening and confirmatory assays as defined per protocol (Supplemental Table 2) and sampling for blood cultures. Conventional radiography (e.g., chest and spine) and abdominal ultrasound could be requested whenever clinically indicated.

When there was no clinical contraindication, cerebrospinal fluid (CSF) was systematically obtained by lumbar puncture and submitted to a set of basic and confirmatory analyses, either on site or in external laboratories (Supplemental Table 2). Lumbar puncture was absolutely contraindicated in case of unarousable coma or rapid deterioration of consciousness, hemodynamic or respiratory instability, papilledema on ophthalmoscopy, bleeding diathesis, skin infection at puncture site, or severe vertebral deformities. In case of responsive and nonrapidly progressive altered state of consciousness or focal neurological deficits, lumbar puncture was performed on a case-by-case decision basis, whenever an underlying CNS infection was suspected.

Blood and CSF samples were transported to Kinshasa to perform bacterial cultures in the Unit of Bacteriology of the “Institut National de Recherche Biomédicale” (INRB) and molecular testing for tuberculosis in the Médecins Sans Frontières-supported “Centre Hospitalier de Kabinda”, as well as shipped to the Institute of Tropical Medicine (ITMA), Antwerp, Belgium, for additional investigations and quality control, as per protocol (Supplemental Table 2). Results of the tests performed in external laboratories were not available for case management, except those realized in Kinshasa, which were communicated by phone.

Based on the clinical and laboratory findings immediately available or secondarily obtained, all study participants were treated according to local therapeutic protocols, with advice from expert clinical consultants when needed. The clinical course and response to specific treatment were followed up prospectively during the hospitalization and later on. The final outcome was assessed 3 months after hospital discharge, and again after 6 months for patients with chronic conditions (tuberculosis, HIV, HAT, epilepsy, and psychosis) or with neurological sequelae at 3 months. Possible outcomes at 6 months were death, cure, stabilized chronic disease, moderate or severe neurological sequelae, and loss to follow-up. Chronic diseases were defined as conditions stabilized at 6 months, but for which a complete cure could not be ascertained at that moment. Neurological sequelae were considered as moderate if daily activities were possible but as impaired and severe if the patient could not perform them anymore or remained bedridden.

### Ethics statement.

The study protocol, the informed consent forms and all key study-related documents were approved by the Institutional Review Board of the ITMA and by the Ethical Committees of the University of Antwerp, Belgium, and of the Public Health School of Kinshasa, DRC. Written informed consent was obtained from each participant or from his/her legal representative for those of minor age (< 18 years) or in case the neurological condition would not allow for adequate decision. For minors from 12 to 18 years, in addition to the parental consent, informed assent was also checked, according to the DRC law. In case of illiterate patient/guardian, an independent witness was present during the consent interview and countersigned the consent form. Patients with neurological disorders who refused to participate in the study were treated in the same way as the study participants and at the cost of the study (to avoid that free treatment of study participants only would influence the consent). The study was monitored according to the standards of the Good Clinical (Laboratory) Practice guidelines, under supervision of the ITMA Clinical Trials and Tropical Laboratory Units.^19^ The study was registered at clinicaltrials.gov under the identifier NCT01589289.

### Data management and statistical analysis.

Clinical and laboratory data collected at the study site were copied and double-entered at the INRB, Kinshasa, under the ITMA supervision for data cleaning and accuracy check. Statistical analyses were performed using Stata 14 software. Frequencies of the main etiologies (primary study endpoints) were reported as proportions of the whole study cohort. A bivariate analysis was performed to determine the factors associated with mortality. Those found with a statistically significant association (*P* value < 0.05) were entered in the multivariate model to identify the independent predictors of death.

## RESULTS

### Baseline characteristics and outcome.

A total of 351 patients were enrolled from September 2012 to June 2014, and the follow-up period lasted to January 2015. This number corresponds to approximately 20% of all cases referred by the general practitioners for evaluation by the study neurologist. The number of screened patients could not be precisely retrieved because the referral load was overwhelming at some moments. The main reasons for noneligibility were age of 5 years and below, recent physical trauma, and sequelae of a past event.

Baseline epidemiological and clinical characteristics of the study participants are presented in Table 1. Males accounted for 46%, and 51.5% belonged to the age group 19–49 years. The median distance from patients’ homes to the study hospital of Mosango was 36 km, and 33% of study participants came from outside the catchment area (another health district).

**Table 1:** Baseline characteristics and clinical outcome of patients admitted for neurological disorders (*N* = 351) to the rural hospital of Mosango, province of Kwilu, DRC

	*N* = 351
Epidemiological data	
Male sex, *n* (%)	163 (46.4)
Median age, year (Q1–Q3)	40 (16–64)
≤ 18 years, *n* (%)	58 (16.5)
19 to 49 years, *n* (%)	181 (51.5)
≥ 50 years, *n* (%)	112 (31.9)
Median distance to HGR of Mosango, km (Q1–Q3)	36 (12–125)
Residence outside catchment area, *n* (%)	116 (33.0)
Clinical features	
Main neurological presenting symptoms/signs,* *n* (%)	
Severe headache, *n* (%)	199 (56.7)
With other symptoms/signs of meningism, *n* (%)	38 (10.8)
Gait/walking disorders, *n* (%)	97 (27.6)
Epileptic seizure, *n* (%)	87 (24.8)
Focal sensory-motor deficit and/or cranial nerve lesion, *n* (%)	86 (24.5)
Cognitive decline and/or behavior disturbance, *n* (%)	74 (21.0)
Altered state of consciousness, *n* (%)	54 (15.4)
Change in sleep pattern, *n* (%)	51 (14.5)
Presence of nonneurological symptoms/signs, *n* (%)	135 (38.5)
Fever (reported and/or observed), *n* (%)	101 (28.8)
Respiratory and/or ear-nose-throat symptoms, *n* (%)	70 (20.0)
Abdominal/digestive symptoms, *n* (%)	37 (10.5)
Median duration of main symptoms, days (Q1–Q3)	21 (3–181)
Contact with primary care facility before admission, *n* (%)	164 (47)
Transferred with referral document, *n* (%)	79 (22.5)
Exposure to treatment before admission, *n* (%)	149 (42.4)
Antibiotic alone, *n* (%)	49 (13.9)
Antimalarial alone, *n* (%)	41 (11.7)
Both antibiotic and antimalarial, *n* (%)	59 (16.8)
Past medical history, *n* (%)	134 (34.4)
Tuberculosis, *n* (%)	28 (7.9)
Psychiatric illness, *n* (%)	19 (5.4)
Human African trypanosomiasis, *n* (%)	12 (3.4)
Concomitant medical condition, *n* (%)	57 (16)
Arterial hypertension, *n* (%)	38 (10.8)
Diabetes mellitus (insulin-dependent), *n* (%)	16 (4.5)
Others, *n* (%)	3 (0.9)
Outcome	
Cure, *n* (%)	109 (31.1)
Stabilized chronic conditions,† *n* (%)	97 (27.3)
Moderate or severe neurological sequelae, *n* (%)	76 (21.6)
Lost to follow-up, *n* (%)	40 (11.4)
Death, *n* (%)	29 (8.2)

DRC = Democratic Republic of Congo; HGR = “Hôpital Général de Référence”; Q1–Q3 = quartiles 25th and 75th.

* Number (%) of cases with presence of only one presenting symptom/sign at presentation: 100 (28.4%); of two symptoms/signs: 159 (45.2%); of three symptoms/signs or more: 92 (26.2%).

† Stabilized chronic conditions included diseases for which definitive cure could not be ascertained after 6 months of follow-up but which were clinically controlled without sequelae. This group included human African trypanosomiasis (*N* = 7), tuberculosis (*N* = 5), HIV infection (*N* = 5), epilepsy (*N* = 57), psychosis (*N* = 12), dementia (*N* = 6), and arterial hypertension and/or diabetes (*N* = 5).

Most participants (71.4%) had more than one neurological symptoms/signs at presentation, in various combinations (Table 1). Severe headache was reported by the majority of patients (199, 57.3%). Other frequent clinical presentations were gait/walking disorders (97, 27.6%), epileptic seizure (87, 24.8%), sensory-motor focal deficit (86, 24.5%), and cognitive decline/abnormal behavior (74, 21%); 15.4% of the cases had an alteration of consciousness at admission. Associated nonneurological symptoms and signs were present in 38.5% of the recruited patients, including fever in 28.8%.

The delay before admission was rather long, with a median duration of symptoms/signs of 3 weeks. Forty-seven percent of the study participants had attended another health facility before admission (but less than half of them were directly referred with an accompanying document). Antibiotics or antimalarials had been reportedly administered to 42.4% patients, 17% having received both.

The most frequent past medical histories were tuberculosis (28, 7.9%), psychiatric illness (19, 5.4%), and HAT (12, 3.4%). Arterial hypertension and/or insulin-dependent diabetes mellitus were present in about 15% of the patients.

### Main diagnoses.

The CNS infections listed as the priority conditions accounted for 63 (17.9%) of the 351 cases (Table 2). Unspecified meningoencephalitis and bacterial meningitis were the most frequent diagnoses in this group (33, 9.4% together), followed by second stage HAT and HIV-related neurological disorders (10, 2.8% for each diagnosis). Diagnostic investigations for this subset of etiologies are detailed in Table 2.

**Table 2:** Main etiological or syndromic diagnoses of neurological disorders at the rural hospital of Mosango, province of Kwilu, DRC

Main diagnoses	*N* = 351
Priority conditions/infections of the central nervous system*	63 (17.9)
Unspecified meningoencephalitis	19 (5.4)
Bacterial meningitis with specified pathogen	14 (4.0)
due to *Neisseria meningitidis*	12 (3.4)
HIV and related opportunistic infections	10 (2.8)
Second-stage human African trypanosomiasis	10 (2.8)
Cerebral malaria	5 (1.4)
Tuberculosis of the central nervous system	4 (1.1)
Neurosyphilis	1 (0.3)
Other local/systemic infections with neurological manifestations	60 (17.1)
Upper/lower respiratory tract infection	18 (5.1)
Undifferentiated febrile illness (viral or bacterial)	17 (4.6)
Uncomplicated malaria	10 (2.8)
Spinal tuberculosis	9 (2.5)
Tetanus	3 (0.8)
Bacteremia	3 (0.8)
Noncommunicable neurological illnesses/syndromes	162 (46.1)
Epilepsy	61 (17.3)
Cerebrovascular accident	23 (6.6)
Neuropathic syndromes	19 (5.4)
Degenerative neurological diseases	18 (5.1)
Dementia	9 (2.6)
Extrapyramidal disorders	6 (1.7)
Motor neuron disorders	3 (0.8)
Myelopathic syndrome	12 (3.4)
Headache syndrome, unspecified	9 (2.5)
Spondylarthropathy, unspecified	7 (1.9)
Vestibular syndrome	6 (1.7)
Migraine	4 (1.1)
Space-occupying lesion	3 (0.8)
Psychiatric disorders	56 (16.0)
Anxiety depression	42 (12.0)
Psychosis	14 (3.9)
Metabolic or vascular diseases with neurological manifestations	10 (2.8)
Arterial hypertension	4 (1.1)
Hyperglycemia	2 (0.5)
Hypoglycemia	2 (0.5)
Temporalis arteritis	2 (0.5)

DRC = Democratic Republic of Congo; HIV = human immunodeficiency virus. All results are expressed as n, (%).

* Diagnosis of the priority conditions was obtained with the following investigations (see also Supplemental Table 2): *unspecified meninogoencephalitis* (presence of > 5 white blood cells (with neutrophil or lymphocyte predominance) in cerebrospinal fluid (CSF) and no etiological pathogen demonstrated (*N* = 19); *bacterial meningitis*: positive culture (*N* = 4); positive Gram stain in cerebrospinal fluid with confirmation by Pastorex antigen test (*N* = 7), or not (*N* = 2); positive Pastorex antigen test only (*N* = 1); *human African trypanosomiasis*: demonstration of trypanosomes in CSF only (*N* = 4), in blood and CSF (*N* = 4), in blood, CSF and lymph node (*N* = 2); *HIV-related neurological disorders*: HIV infection confirmed by three successive positive rapid diagnostic tests, with unspecified meningoencephalitis (*N* = 4), cryptococcal meningitis (*N* = 3), or cerebral toxoplasmosis (*N* = 3); *cerebral malaria*: signs of cerebral dysfunction and blood smear positive for *Plasmodium falciparum* trophozoites + and exclusion of other etiologies (*N* = 5); *tuberculosis of central nervous system*: positive Ziehl stain and GeneXpert in CSF (*N* = 1), positive GeneXpert in CSF only (*N* = 2), and abnormal CSF and demonstrated pulmonary tuberculosis (*N* = 1); and *neurosyphilis*: positive rapid plasma reagin and *Treponema pallidum* passive particle agglutination in blood and positive venereal disease research laboratory test in CSF (*N* = 1).

Other focal/systemic infections with neurological manifestations were confirmed or strongly suspected in an additional 60 cases (17.1%) with respiratory tract infection (sinusitis, otitis, and pneumonia), undifferentiated febrile illness, uncomplicated malaria, and spinal tuberculosis as leading diagnoses in this group. No cases of rabies, poliomyelitis, or leprosy were observed during the study period.

Neurological noncommunicable syndromes were diagnosed in approximately half of all patients, with an important burden of epilepsy (61, 17.3%) and cerebrovascular accidents (23, 6.6%). Neuropathic and myelopathic syndromes as well as degenerative neurological diseases were observed with frequencies ranging from 3% to 5%. Other noncommunicable disorders are listed in Table 2.

Psychiatric disorders were seen in 56 cases (16% of the total load). Of note, several metabolic and vascular diseases (diabetes and arterial hypertension) were occasionally diagnosed in the study participants as the likely etiology of their neurological manifestations.

### Frequencies of the priority and other leading conditions according to neurological symptom/sign of presentation.

Table 3 summarizes the frequencies of the main neurological diagnoses according to the different clinical presentations, in other words the pretest probability of a given neurological disease according to the main presenting symptom/sign in this setting. Bacterial meningitis, unspecified meningoencephalitis, HIV-related neurological disorders, cerebrovascular accident, and epilepsy accounted all together for more than half of the patients presenting with altered consciousness, whereas psychiatric disorders and HAT did so for change in sleep pattern. Two third of patients admitted for epileptic seizures were eventually diagnosed with epilepsy. Cognitive decline and behavior disturbance were mainly seen in people with psychiatric disorders and epilepsy. In patients with focal deficits, cerebrovascular accident was by far the main clinical diagnosis, but unspecified meningoencephalitis and HIV-related neurological disorders were rather frequent as well. The leading causes of gait/walking disorders included cerebrovascular accident, spinal tuberculosis, and unspecified meningoencephalitis.

**Table 3:** Frequency of the priority and other leading conditions according to the main symptoms/signs of presentation in the patients with neurological disorders at the rural hospital of Mosango, province of Kwilu, DRC

	Altered consciousness (*N* = 54)	Change in sleep pattern (*N* = 51)	Cognitive decline/behavior disturbance (*N* = 74)	Epileptic seizure (*N* = 87)	Headache +/− meningism (*N* = 199)	Focal deficits/cranial nerve lesion (*N* = 86)	Gait/walking disorders (*N* = 97)
Unspecified meningoencephalitis (*N* = 19)	10 (18.5)	3 (6)	6 (8)	8 (9)	14 (7)	9 (10.5)	8 (8)
Bacterial meningitis (*N* = 14)	10 (18.5)	1 (2)	2 (2.5)	–	13 (6.5)	–	1 (1)
Second stage HAT (*N* = 10)	3 (5.5)	6 (12)	7 (9.5)	–	5 (2.5)	2 (2.5)	5 (5)
HIV-related neurological disorders (*N* = 10)	4 (7.5)	1 (2)	3 (4)	2 (2.5)	6 (3)	6 (7)	4 (4)
Tuberculosis of CNS (*N* = 4)	2 (3.5)	–	1 (1.5)	–	4 (2)	1 (1)	–
Cerebral malaria (*N* = 5)	1 (2)	–	1 (1.5)	3 (3.5)	3 (1.5)	–	1 (1)
Spinal tuberculosis (*N* = 9)	–	–	1 (1.5)	–	2 (1)	5 (6)	9 (9.5)
Epilepsy (*N* = 61)	4 (7.5)	2 (4)	16 (21.5)	59 (68)	28 (14)	2 (2.5)	1 (1)
Psychiatric disorders (*N* = 56)	1 (2)	18 (35.5)	20 (27)	–	43 (21.5)	3 (3.5)	2 (2)
Cerebrovascular accident (*N* = 23)	6 (11)	1 (2)	2 (2.5)	2 (2.5)	5 (2.5)	25 (29)	19 (19.5)

CNS = central nervous system; DRC = Democratic Republic of Congo; HAT = human African trypanosomiasis; HIV = human immunodeficiency virus. All results are presented as *n* (%); all % have been rounded at 0.5.

### Outcome.

The rate of loss to follow-up was low (11%) taking into consideration the difficult study conditions. A total of 29 enrolled patients died, corresponding to an overall fatality rate of 8.2%, including 16/29 (55%) within 3 days after admission (Table 1). The case fatality rates were 23.8% (15/63), 11.7% (7/60), and 3.1% (7/228) for the priority conditions, the other focal/systemic infections, and the remaining noncommunicable diseases, respectively. At least 76 patients (21.6% of the total inclusions) had moderate or severe sequelae after 6 months of follow-up, whereas the remaining was considered as clinically cured or stabilized.

The risk factors associated with fatal outcome could be analyzed for 311 patients (88.6%) with complete initial and follow-up data (28 deaths and 283 survivors). Features associated with death in the bivariate analysis were symptom duration < 1 week, prior contact with primary health facilities, prior treatment, and several clinical features at presentation (Table 4). When the multivariate model was applied, the only independent predictors of death were the following clinical features: cachexia (defined as body mass index < 20) with an adjusted odds ratio (aOR) of 8.2 (95% confidence interval [CI]: 3–25.2); neck stiffness, aOR 6.0 (95% CI: 1.5–24); altered consciousness, aOR 4.9 (95% CI: 1.7–14.0); and fever, aOR 3.5 (95% CI: 1.3–9.5).

**Table 4:** Risk factors of death in patients admitted for neurological disorders at the rural hospital of Mosango, province of Kwilu, DRC, and with complete follow-up data (*N* = 311)

	Bivariate analysis				Multivariate analysis	
Variables	Deaths (*N* = 28*)	Survivors (*N* = 283)	OR (95% CI)	*P*	OR	*P*
Epidemiological features						
Male	16 (57.1)	126 (44.5)	1.6 (0.8–4.0)	0.205	–	–
Age (years)						
19–49 years	18 (64.2)	142 (50.2)	2.4 (0.8–7.3)	0.129		
≥ 50 years	6 (21.4)	66 (23.3)	1.7 (0.5–6.3)	0.424		
Distance from hospital > 35 km	14 (50.0)	177 (62.5)	0.5 (0.2–1.3)	0.169	–	–
Residence outside catchment area	8 (28.5)	86 (30.3)	0.9 (0.4–2.2)	0.842	–	–
Duration of symptoms > 1 week	12 (43.0)	190 (67.1)	0.4 (0.2–0.8)	0.013	0.8 (0.2–3.3)	0.733
Prior contact with primary care	22 (78.5)	123 (43.5)	4.7 (1.9–12.1)	0.001	2.3 (0.2–24.6)	0.486
Previous antibiotic/antimalarial treatment	22 (78.5)	106 (37.4)	6.1 (2.4–15.6)	< 0.001	1.7 (0.2–17.7)	0.650
Clinical features						
Altered consciousness	16 (57.1)	32 (11.3)	10.5 (4.5–24.1)	< 0.001	4.9 (1.7–14.0)	0.003
Gait/walking disorders	6 (21.4)	77 (27.2)	0.7 (0.3–2.0)	0.511	–	–
Epileptic seizure	7 (25.0)	73 (26.0)	0.9 (0.4–2.3)	0.927	–	–
Headache	11 (39.2)	134 (47.3)	0.7 (0.3–1.6)	0.416	–	–
Change in sleep pattern	2 (1.0)	42 (15.0)	0.4 (0.1–2.0)	0.277	–	–
Neck stiffness (flexion and rotation)	13 (46.4)	71 (25.1)	9.3 (3.0–31.4)	< 0.001	6.0 (1.5–24.0)	0.011
Focal sensory-motor deficit/cranial nerve lesion	5 (18.0)	68 (24.0)	0.7 (0.2–2.0)	0.465	–	–
Cognitive decline/behavior disturbance	7 (25.0)	59 (21.0)	1.3 (0.5–3.1)	0.609	–	–
Cachexia (body mass index < 20)	10 (36.0)	24 (8.0)	6.0 (2.5–14.4)	< 0.001	8.2 (3.0–25.2)	0.001
Dehydration	16 (57.1)	42 (15.0)	8.0 (3.4–17.3)	< 0.001	1.8 (0.4–8.4)	0.438
Fever (reported/observed)	16 (57.1)	33 (17.0)	10.1 (4.4–23.2)	< 0.001	3.5 (1.3–9.5)	0.014
Vomiting	5 (18.0)	17 (6.0)	3.24 (1.1–10.0)	0.027	0.1 (0.0–3.0)	0.192
Laboratory features						
White blood cell > 10,000/µL†	9/21 (42.8)	26/221 (11.7)	5.6 (2.1–14.6)	< 0.001	–	–

CI = confidence interval; DRC = Democratic Republic of Congo; OR = odds ratio. All results are *n* or *n*/*n* (%), except otherwise specified. Only features associated with death in bivariate analysis (*P* value < 0.05) were entered in the multivariate model.

* One patient died before the full neurological and laboratory assessment could be performed, and therefore is excluded from this analysis.

† This parameter has not been entered in the multivariate analysis, because there were too many missing data (because of a temporary technical problem).

## DISCUSSION

In this clinical study investigating neurological disorders among children > 5 years and adults admitted in a general referral hospital of rural DRC, we observed that the pattern of clinical presentations was extremely diverse and complex. Both infectious and noncommunicable diseases were frequent, with infections of the CNS, epilepsy, psychiatric disorders, and cerebrovascular accidents accounting altogether for 60% of the total case load. The mortality and morbidity were considerable, in particular for the subset of CNS infections, and fatal outcome was predicted by the clinical presentation rather than by epidemiological characteristics in this setting.

The strength of this large prospective study is that it has investigated with the best diagnostic methods that could be made available the etiological spectrum of recent and/or ongoing neurological disorders as they present in a rural African hospital. It provides a rather comprehensive and representative picture of the neurological disorders requiring hospitalization in this deprived region of Africa, including a reliable description of their clinical outcome. The study has on the other hand obvious limitations. First, although our investigations went far beyond the local standard of care, diagnostic uncertainty remained important in the absence of neurophysiological tests (such as electroencephalogram or electromyography), neuroimaging, or molecular microbiological assays, even if all these tools do not necessarily allow a full appraisal of all etiologies.^20,21^ We tried to bridge this gap by using predefined clinical definitions of diseases and submitting all cases to an expert consensus panel, so we consider that the resulting diagnostic ascertainment was internally consistent and robust. Second, we missed in this study an unknown proportion of cases who attended only the first-line facilities or other pathways of care, as well as those most severe and acute cases who could not reach the study hospital in time. Finally, the single center design, because of evident logistical constraints, somehow limits the generalizability of the findings, although we believe that the hospital where this study took place is fairly representative of most of those of this subregion.

As expected, headaches, epileptic seizure, and focal deficits were the most frequently observed neurological disorders. However, other neurological syndromes such as gait/walking disorders and cognitive decline/behavior disturbances were also surprisingly frequent. Moreover, various neurological symptoms and signs were present concomitantly. These observations are important for medical faculties that have to adequately prepare general practitioners and nurses for the complex management of neurological disorders in remote African areas. In our cohort, altered consciousness was less frequent than in the largest prospective series to date in North Tanzania,^22^ where this syndrome was the cause for more than half of the neurological admissions. The difference is likely explained by the exclusion of post-traumatic pathology in our study, the low prevalence of HIV infection in our cohort (although it was not tested in the Tanzanian study), and by the more general criteria of inclusion we used to capture a maximum of neurological disorders since their earliest stages.

About 20% of the patients with neurological disorders suffered from CNS infections that were robustly confirmed by a panel of reference tests. Previous studies provided contradictory results and reported either lower or higher prevalence estimates of infections that likely depended on different periods,^5,6^ settings,^8^ inclusion criteria,^22^ or diagnostic tests.^9,10^ Bacterial meningitis, largely preventable by vaccination, and unspecified meningoencephalitis accounted for up to 10% of the total case load, even in the older population of our study. By contrast, and not surprisingly, cerebral malaria was rather infrequent in this hyperendemic area, where severe malaria disproportionally affects children less than 5 years. The frequency of HAT was lower than initially expected, reflecting the major progress in disease control recently achieved in DRC.^23^ Similarly, HIV infection was not very frequent in the study population, corresponding to its continuing rather low general prevalence in DRC.^24^ This had of course a major impact on the observed pattern of etiologies, when compared with high HIV-burden areas such as southern Africa, where tuberculosis and HIV-associated cryptococcal disease together can account for up to 70% of identified pathogens in similar populations.^25,26^

Noncommunicable diseases accounted for at least half of the admissions in this nonspecialized rural setting serving a mixed general population, as observed elsewhere.^10^ Epilepsy was by far the leading single diagnosis, underlining the particular attention this condition deserves in terms of appropriate management, availability of drugs,^27^ training needs and research targeting, among others, the potential contribution of *Taenia solium* neurocysticercosis.^28,29^ Similarly, cerebral complications or manifestations of metabolic diseases were also common, highlighting again the pressing need to design, at the grass-root, community, national, and global levels, field-adapted interventions that will address this noncommunicable epidemic. Also, the substantial proportion of psychiatric disorders reminds us the importance of developing strong global mental health programs,^30^ preferably community based, adapted to populations affected by social instability and extreme poverty in regions where psychiatric services are almost nonexistent. Finally, additional research and innovative technologies are required to address the large “residual” amount of unspecified neurological syndromes with unknown etiologies.

Many cases had an adverse outcome. Death rate of the CNS infections was particularly striking despite the best care that could be provided in this harsh environment. Fatal outcome was mainly predicted by clinical features of advanced and severe disease at presentation (altered consciousness, cachexia, fever, etc.) rather than epidemiological risk factors such as the distance to hospital or age. This stresses again that early diagnosis in the field is key and has more than ever to rely on a careful and soundly taught medical examination, supported by the judicious use of appropriate point-of-care discriminative tools.^14^

In conclusion, a large subset of neurological disorders in rural Africa may be preventable and treatable. Therefore, it is of paramount importance that concerted efforts by caregivers, researchers, and policy makers converge to make an accurate diagnosis possible via innovative field technology and access to existing tools and diagnostic infrastructure, as well as management effective through appropriate problem-based training and provision of adequate resources. In addition, the results presented in this article deliver useful information for the recently worldwide adopted sustainable development goals that put special emphasis on global mental health disorders.

## Supplementary Material

Supplemental Table.
