# Supplementary material for: Clinical Spectrum, Etiology, and Outcome of Neurological Disorders in the Rural Hospital of Mosango, the Democratic Republic of Congo
Source: Am J Trop Med Hyg. 2017 Aug 14;97(5):1454–60. doi: 10.4269/ajtmh.17-0375 (PMC5817781; doi:10.4269/ajtmh.17-0375)
Supplement: Supplementary file 1 [file tpmd170375.SD1.pdf]

Supplemental Table 1

Definition, description, and rationale of the inclusion and exclusion criteria of the NIDIAG study

Inclusion criteria

1) Altered state of consciousness
Pragmatically divided into four stages:
- confusion (“qualitative alteration”): alert but inadequate in simple answers; sometimes talking nonsense; disoriented in place and/or time and/or person (Glasgow scale 14/15)
- somnolence: sleepy, abnormally slow in formulating idea; lethargic, but easily wakened with slight stimulation
- stupor: difficult to wake up, only with robust (but nonpainful) stimulation
- coma: reactive to pain or not (Glasgow scale below eight)

Most of these symptoms (mainly reported by relatives) are usually acute, but may last for weeks or even months for some diseases such as human African trypanosomiasis, neurosyphilis, etc.

2) Changes in sleep pattern
Patient presents with night insomnia, although sleep was normal previously. He/she may also be sleepy, lethargic during the day. There is no specific restriction regarding the duration of such symptoms before consulting (weeks or months), provided that the symptom is still present at screening. The symptom may exceed 1 year as seen in human African trypanosomiasis.

3) Cognitive decline
Patient develops cognitive disturbances, not present previously, such as memory impairment, up to dementia. Most of the time, these symptoms will be obtained by collateral history taking. There is no specific restriction regarding the duration of such symptoms before consulting as far as they are still present at evaluation for inclusion.

4) Changes in personality/behavior
Changes in patient’s personality, either rapid and/or progressive, are observed by the family and are a source of concern (abnormal euphoria, severe depression, agitation, strange behaviors, etc.). Same remark as for criteria 3 regarding duration of symptoms

5) Recent (< 2 weeks) epileptic seizure(s)
Sudden onset of stiffness and/or jerking of arms and legs, localized or generalized (with loss of consciousness), with the last seizure having occurred within 2 weeks before screening, in a patient who never experienced this problem at 5 years of age or below (see exclusion criteria). Tongue bite and incontinence of urine and/or feces may be present. Furthermore, the patient may have experienced injuries during the actual seizure.

6) Recent, severe, and progressive headache
Patient complains of headache which remains present every day, does not resolve with symptomatic treatment and even increases in severity (with or without other symptoms), and interfering with normal daily activities.

7. Meningism
Classic signs of meningism include severe and worsening headache (relieved by lying down), nuchal rigidity (pain and neck stiffness with rotation usually less affected), and signs of intracranial hypertension or irritation (nausea/vomiting; phono-photophobia). This syndrome may be present for hours, days, or weeks provided that it is still ongoing at evaluation for inclusion.

8) New onset cranial nerve lesions
New onset appearance of one or more abnormal symptoms/signs, mostly localized on head or face such as hearing or visual impairment, eye movement abnormalities, pupil abnormalities, facial palsy, dysphagia or dysarthria, etc. suggesting an underlying cranial nerve lesion. “New onset” means arbitrarily occurring/starting within a year before screening, but exceptions (longer symptom duration) are possible if considered relevant.

9) New onset sensory-motor deficits or other focal neurological signs (e.g., ataxia, dystonia)
Patient complains of new onset sensory or motor disturbance (e.g., hypoesthesia, paresis, paralysis) causing problems to walk, to grasp or to perform current physical activities. “New onset” means arbitrarily occurring/starting within a year before screening, but exceptions (longer symptom duration) are possible if considered relevant.

10) New onset neurological gait/walking disorders (e.g., spastic/ataxic/paretic gait)
Patient has difficulty he/she did not experience before to stand up or walk because his balance, coordination, motor strength or sensation are impaired. “New onset” means arbitrarily occurring/starting within a year before screening, but exceptions (longer symptom duration) are possible if considered relevant. Of note, patients will not be included if the gait/walking disorder has an obvious orthopedic origin.

Exclusion criteria

1) Children of 5 years or below: Morbidity profile of neurological disorders is very different in younger children and has been frequently studied. Young children do not represent the target group for this study.
2) Neurological symptom/sign clearly related to recent trauma (accident, etc.): obvious nonmedical etiology.
3) Neuropsychiatric symptom clearly related to a recent social/familial stress: symptoms due to evident poststress reactive depression or psychosis according to the investigator. In case of doubt, include provided that at least one inclusion criterion is present.
4) Neurological symptom as sequelae of an old, well-established neurological event (e.g., stroke, etc.): the study is not aimed/designed at exploring long-term neurological sequelae.
5) First (epileptic) seizure at 5 years or below: in such cases, sequel of an early onset neurological event is much more likely, and febrile seizures as a confounder for epilepsy are common.
6) Patients unable in the physician’s opinion or unwilling to comply with the study requirements (for example refusing hospital admission or scheduled clinical examinations).

Supplemental Table 2

Composite case definitions of priority conditions (confirmed and probable)

Disease	Reference test(s)	Location Reference test
Human African trypanosomiasis (HAT), second stage	**Confirmed**:<br>Demonstration of trypanosomes in any relevant body fluid: cervical lymph node aspirate (LNA), if present; blood using mini Anion exchange centrifugation technique (mAECT); CSF using modified single centrifugation (MSC)<br>Diagnosis of second-stage HAT if trypanosomes detected in CSF, or both of the following conditions met: trypanosomes detected in any body fluid and CSF white blood cell count > 5/μL	Study site ("Hôpital Général de Référence" of Mosango): microscopy
Cerebral malaria	Diagnosis of probable cerebral malaria based on the WHO 2,000 case definition of severe falciparum malaria (*Trans R Soc Trop Med Hyg* 2000;94(Suppl 1): S1–90), requiring the following:<br>1) Patient presents with impaired consciousness or with repeated convulsions (> 2 episodes within 24 hours)<br>2) Presence of *Plasmodium falciparum* asexual parasites in peripheral blood<br>3) No additional coinfections other than HIV are identified that could explain the patient's symptoms<br>4) Normal CSF findings (meaning white blood cell count below 5/μL)<br>5) Dramatic neurological improvement to specific antimalarial treatment<br>Note 1: *Cerebral malaria is operationally considered as probable because the causal relationship between presence of parasites and neurological symptoms cannot be completely ascertained in highly endemic settings, where asymptomatic parasitemia is frequent*<br>**Note 2: uncomplicated malaria**<br>Cases not fulfilling the definition of cerebral malaria (see previous definition) in whom neurological symptoms and signs are likely attributable to malaria (presence of trophozoites of any species at blood microscopy, clinical response to antimalarials, and absence of other etiology) will be categorized as **"**uncomplicated malaria**"** and reported in the group "systemic infections" (not as a priority condition in this study group older than 5 years)	Study site ("Hôpital Général de Référence" of Mosango): microscopy
Bacterial meningitis	**Confirmed**	Study site ("Hôpital Général de Référence" of Mosango): sampling for culture, CSF microscopy, Gram staining on CSF
Unspecified meningoencephalitis	Clinically compatible case with isolation of a bacterial species from either CSF or blood<br><br>Clinically compatible case with pleocytosis in CSF (> 5 white blood cells/μL) and positive CSF Gram stain and/or positive Pastorex Meningitis antigen assay (Bio-Rad, Hercules, CA)<br>**Unspecified meningoencephalitis: probable**<br>Cases with clinical features of meningoencephalitis and pleocytosis at CSF examination but without demonstration of etiological pathogen are diagnosed as **"**unspecified meningoencephalitis**"** (whatever the clinical response to antibiotics)<br>Note: *Because such cases always require antibiotics in clinical practice, they are part of the priority conditions*	Institute of Tropical Medicine, Antwerp, Belgium: Pastorex Meningitis antigen assay (Bio-Rad, Hercules, CA)

(continued)

Supplemental Table 2

Continued

Disease	Reference test(s)	Location Reference test
Tuberculosis of central nervous system (CNS)	Uniform case definition for confirmed and probable cases, as proposed by Marais et al. in "Tuberculous meningitis: a uniform case definition for use in clinical research (*Lancet Infect Dis* 2010;10(11): 803–812), in settings with no neuroimaging<br>Note: *This case definition is selected because it addresses longstanding concerns in studies of tuberculous meningitis about comparability between studies and suitability for low-resource settings. It uses a scoring system and relies to a large extent on the ability to isolate Mycobacterium tuberculosis and/or exclude other diagnoses*<br>**Confirmed:**<br>Clinically compatible illness and acid-fast bacilli seen by Ziehl staining in the CSF, or a CSF culture positive for MTB (identified by commercial nucleic acid amplification test (GeneXpert) performed on a MGIT culture bottle with bacterial growth)<br>**Probable:**<br>Clinically compatible illness and a total diagnostic score of ≥ 10 (see previous reference) and exclusion of alternative diagnoses	Study site ("Hôpital Général de Référence" of Mosango): Ziehl staining on CSF (and sputum or abscess if clinically indicated); inoculation of CSF in MGIT tubes
Neurosyphilis	**Confirmed**:	Study site ("Hôpital Général de Référence" of Mosango): Macro-Vue RPR Card Tests (Becton, Dickinson and company, NJ)
	Clinical compatible illness and<br>• a reactive nontreponemal (Rapid Plasma Reagin, RPR) and treponemal test (*Treponema pallidum* passive particle agglutination, TPPA) in serum for syphilis and<br>• a reactive Venereal Disease Research Laboratory (CSF-VDRL) test in cerebrospinal fluid (CSF); sensitivity of 50% and specificity of about 100% for diagnosing neurosyphilis<br>**Probable**:<br>• a reactive RPR and TPPA test in serum (see above)<br>• a negative CSF-VDRL<br>• elevated CSF protein or white blood cell > 5/μL, with no alternative diagnosis and clinical response to penicillin treatment<br>Note: *The sequence of testing is as follows: serum RPR performed to all study participants, at the study site. Reactive sera to be submitted to serum TPPA test confirmation. If TPPA positive, determination of VDRL in CSF*	Institute of Tropical Medicine, Antwerp, Belgium: TPPA (and VDRL on CSF)
HIV-related neurological disorders	**Confirmed HIV infection**	Study site ("Hôpital Général de Référence" of Mosango): HIV-RDTs in blood; Indian ink on CSF; CrAg LAT in CSF or serum
	Following the HIV control program in DRC, HIV infection confirmed if all three following HIV rapid diagnostic tests (RDTs) sequentially performed are all positive: 1) Determine HIV-1/2 (Alere, Waltham, MA); 2) Uni-Gold HIV (Trinity Biotech, Bray, Wicklow, Ireland) and 3) DoubleCheckGold HIV 1&2 (Alere, Waltham, MA)<br>***HIV-related cryptococcal meningitis: confirmed***<br>Clinically compatible illness and any positive results from:<br>• Indian ink examination on CSF	Unit of bacteriology, "Institut National de Recherche Biomédicale", Kinshasa, DRC: blood and CSF cultures

(continued)

Supplemental Table 2

Continued

Disease	Reference test(s)	Location Reference test
	• Cryptococcal cultures in blood or CSF<br>• Reference Cryptococcal Antigen (CrAg) Latex Agglutination test (LAT) in serum or CSF<br>***HIV-related toxoplasmic encephalitis: probable***<br>Clinically compatible illness characterized by focal neurological signs with or without fever, and with dramatic clinical improvement following a specific treatment against cerebral toxoplasmosis (trimethoprim-sulfamethoxazole)<br>***HIV-related unspecified meningoencephalitis: probable***<br>Diagnosis of unspecified meningoencephalitis (as described previously here) in a patient with confirmed HIV infection<br>***HIV-related neurological disorder: probable***<br>Illness with the presence of neurological symptoms/signs in a patient with confirmed HIV infection, and not fulfilling any of the three case definitions here above	

CNS = central nervous system; CSF = cerebrospinal fluid; DRC = Democratic Republic of Congo; MGIT = Mycobacteria Growth Indicator Tube; MTB = *Mycobacterium tuberculosis*; HIV = human immunodeficiency virus; RDT = rapid diagnostic test; WHO = World Health Organization. Bold was used to make/highlight/structure the differences between definitions of “confirmed” and “probable” infections, and the (sub)diagnoses related to HIV infection in this section.

Supplemental Table 3

Additional clinical case definitions for the NIDIAG study on neurological disorders in DRC, Neurological and psychiatric illnesses

Diseases/syndromes	NIDIAG clinical case definition	References/Sources
Anxiety/depression	Symptoms lasting for several days, associated with anxiety and worries, and characterized by sleep disturbances, restlessness, fatigue, difficult concentration, irritability, or muscle tension. Resolution either spontaneous or with anxiolytic treatment	Diagnostic and Statistical Manual of Mental Disorders (4th ed.; DSM-IV TR; American Psychiatric Association, 2000): dsm.psychiatryonline.org/doi/pdf/10.1176/appi.books.9780890420249.dsm-iv-tr
Cerebellar syndrome	Ataxia (reeling, wide-based gait), dysdiadochokinesia (inability to perform rapid alternating movements), dysmetria (inability to control range of movement) and tremor (intention tremor)	NIH: National Institute of Neurological Disorders and Stroke http://www.ninds.nih.gov/disorders/ataxia/ataxia.htm<br>Ropper AH, Samuels MA, Klein JP. Adams & Victor's Principles of Neurology. 10th edition. 2014, China. Chapter 5. Ataxia and Disorders of the Cerebellar Function; 81<br>Daroff RB, Fenichel G, Jankovic J, Mazziotta JC. Bradley's Neurology in Clinical Practice. Principles of Diagnosis and Management. 7th edition. 2016, Philadelphia. Chapter 22. Ataxic and Cerebellar disorders. Subramony SH, Xia G; 217
Cerebrovascular accident	Acute onset of focal neurological deficit that may correspond to a specific cerebral vascular territory and/or be associated with the absence of infectious symptoms/signs. Usually without further progression of neurological symptoms/signs. Cardiovascular risk factors may be present	An Updated Definition of Stroke for the 21st Century http://stroke.ahajournals.org/content/44/7/2064<br>Ropper AH, Samuels MA, Klein JP. Adams & Victor's Principles of Neurology. 10th edition. 2014, China. Chapter 34. Cerebrovascular Diseases; 778<br>Daroff RB, Fenichel G, Jankovic J, Mazziotta JC. Bradley's Neurology in Clinical Practice. Principles of Diagnosis and Management. 7th edition. 2016, Philadelphia. Chapter 25. Hemiplegia and monoplegia. Misulis KE, Murray EL; 262
Dementia/mental retardation	Impairment of cognition unexplained by physical or acute intracranial pathology, either due to intellectual decline (dementia) or non-achievement of full mental capacities (mental retardation)	Ropper AH, Samuels MA, Klein JP. Adams & Victor's Principles of Neurology. 10th edition. 2014, China. Chapter 21. Dementia, the Amnesic Syndrome, and the Neurology of Intelligence and Memory; 434.<br>Daroff RB, Fenichel G, Jankovic J, Mazziotta JC. Bradley's Neurology in Clinical Practice. Principles of Diagnosis and Management. 7th edition, 2016, Philadelphia. Chapter 7. Intellectual and Memory impairment. Kirshner HS, Ally B; 57
Epilepsy	At least two unprovoked epileptic seizures, unrelated to acute metabolic disorders or withdrawal of alcohol, occurring greater than 24 hours apart without any epileptic seizures before this period	International League Against Epilepsy (ILAE) 2014 http://www.ilae.org/visitors/centre/Definition-2014.cfm.<br>Senanayake N, Roman G. Epidemiology of epilepsy in developing countries. Bull WHO 1993; 71: 247–258
Extrapyramidal disorders	Signs including dyskinesia or akinesia, dystonia, rigor, tremor and other movement disorders such as motor tics, etc. without loss of voluntary movement	Ropper AH, Samuels MA, Klein JP. Adams & Victor's Principles of Neurology. 10th edition. 2014, China. Chapter 4. Abnormalities of Movement and Posture Caused by Disease of the Basal Ganglia; 64.<br>Ropper AH, Samuels MA, Klein JP. Adams & Victor's Principles of Neurology. 10th edition. 2014, China. Chapter 6. Tremor, Myoclonus, Focal Dystonias, and Tics; 92

(continued)

Supplemental Table 3
Continued

Diseases/syndromes	NIDIAG clinical case definition	References/Sources
		Daroff RB, Fenichel G, Jankovic J, Mazziotta JC. Bradley's Neurology in Clinical Practice. Principles of Diagnosis and Management. 7th edition. 2016, Philadelphia. Chapter 23. Diagnosis and Assessment of Parkinson Disease and Other Movement Disorders. Jankovic J, Lang AE; 223
Headache, unspecified	Severe headache as a major complaint (e.g., interfering normal daily activities) but without evidence of systemic (febrile) disease and not meeting the case definitions of other neurological disorders. May include tension type headaches that can be severe at times	Ropper AH, Samuels MA, Klein JP. Adams & Victor's Principles of Neurology. 10th edition. 2014, China. Chapter 10. Headache and Other Craniofacial Pains; 162<br>Daroff RB, Fenichel G, Jankovic J, Mazziotta JC. Bradley's Neurology in Clinical Practice. Principles of Diagnosis and Management. 7th edition. 2016, Philadelphia. Chapter 20. Cranial and Facial Pain. Bartleson JD, Black DF, Swanson JW; 197<br>Diagnosis and Management of Headaches in Young People and Adults (National Institute for Health and Clinical Excellence 2012)
Migraine	Severe unilateral headache of different qualities (e.g., throbbing, and pulsating) often changing sides. Associated symptoms may include nausea, vomiting, and sensitivity to light, sound, or smell as well as focal neurological signs	Ropper AH, Samuels MA, Klein JP. Adams & Victor's Principles of Neurology. 10th edition. 2014, China. Chapter 10. Headache and Other Craniofacial Pains; 162<br>Daroff RB, Fenichel G, Jankovic J, Mazziotta JC. Bradley's Neurology in Clinical Practice. Principles of Diagnosis and Management. 7th edition. 2016, Philadelphia. Chapter 20. Cranial and Facial Pain. Bartleson JD, Black DF, Swanson JW; 197<br>Diagnosis and Management of Headaches in Young People and Adults (National Institute for Health and Clinical Excellence 2012)
Motor neuron disorders	Clinical signs resulting from a lesion of the upper (e.g., spasticity and increased reflexes) or/and lower (e.g., flaccid paresis/paralysis and muscle wasting associated with fasciculations)	NIH: National Institute of Neurological Disorders and Stroke http://www.ninds.nih.gov/disorders/motor_neuron_diseases/detail_motor_neuron_diseases.htm<br>Ropper AH, Samuels MA, Klein JP. Adams & Victor's Principles of Neurology. 10th edition. 2014, China. Chapter 3. Motor Paralysis; 43<br>Daroff RB, Fenichel G, Jankovic J, Mazziotta JC. Bradley's Neurology in Clinical Practice. Principles of Diagnosis and Management. 7th edition. 2016, Philadelphia. Chapter 98. Disorders of Upper and Lower Motor neurons. Fearon C, Murray B, Mitsumoto H; 1484
Myelopathic syndrome	Symptoms/signs associated to inflammatory/infectious/degenerative/compressive damage to the spinal cord either acute (impairment of sensation/flaccid paralysis) or progressive (hyperreflexia + spasticity if cervical or thoracic spinal cord lesion). For definitions of cervical, thoracic and lumbar spinal cord syndromes, see references on the right	Ropper AH, Samuels MA, Klein JP. Adams & Victor's Principles of Neurology. 10th edition. 2014, China. Chapter 44. Diseases of the Spinal Cord; 1181<br>Daroff RB, Fenichel G, Jankovic J, Mazziotta JC. Bradley's Neurology in Clinical Practice. Principles of Diagnosis and Management. 7th edition. 2016, Philadelphia. Chapter 26. Paraplegia and Spinal cord Syndromes. Dobkin BH; 273

(continued)

Supplemental Table 3
Continued

Diseases/syndromes	NIDIAG clinical case definition	References/Sources
Myopathic syndrome	Muscle weakness and/or pain and/or wasting without any sensory impairment in the absence of signs/symptoms of peripheral neuropathies, radiculopathies or motor neuron disease, amongst others	NIH: National Institute of Neurological Disorders and Stroke http://www.ninds.nih.gov/disorders/myopathy/myopathy.htm<br>Ropper AH, Samuels MA, Klein JP. Adams & Victor's Principles of Neurology. 10th edition. 2014, China. Chapter 48. Disease of the Muscles; 1403<br>Daroff RB, Fenichel G, Jankovic J, Mazziotta JC. Bradley's Neurology in Clinical Practice. Principles of Diagnosis and Management. 7th edition. 2016, Philadelphia. Chapter 28. Muscle pain and Cramps. Wang LH, Lopate G, Pestronck A; 296
Neuropathic syndrome	Symptoms/signs related to lesion/compression of single or multiple peripheral nerves. Includes radiculopathies, plexopathies, mono- or polyneuropathies, and polyradiculoneuropathies (= Guillain-Barré syndrome). Cranial neuropathy designates pathology of cranial nerves	NIH: National Institute of Neurological Disorders and Stroke http://www.ninds.nih.gov/disorders/peripheralneuropathy/detail_peripheralneuropathy.htm<br>Ropper AH, Samuels MA, Klein JP. Adams & Victor's Principles of Neurology. 10th edition. 2014, China. Chapter 46. Diseases of the Peripheral Nerves; 1310<br>Ropper AH, Samuels MA, Klein JP. Adams & Victor's Principles of Neurology. 10th edition. 2014, China. Chapter 47. Diseases of the Cranial Nerves; 1391<br>Daroff RB, Fenichel G, Jankovic J, Mazziotta JC. Bradley's Neurology in Clinical Practice. Principles of Diagnosis and Management. 7th edition. 2016, Philadelphia. Chapter 106. Disorders of Nerve Roots and Plexuses. Chad DA, Bowley MP; 1766<br>Daroff RB, Fenichel G, Jankovic J, Mazziotta JC. Bradley's Neurology in Clinical Practice. Principles of Diagnosis and Management. 7th edition. 2016, Philadelphia. Chapter 107. Disorders of Peripheral Nerves. Katirji B; 1791
Psychosis	Presence of one (or more) of the following psychiatric symptoms/signs: delusions, hallucinations, disorganized speech (e.g., frequent derailment or incoherence), grossly disorganized or catatonic behavior, amongst others; good clinical response with neuroleptic medication	Diagnostic and Statistical Manual of Mental Disorders (4th edition; DSM-IV TR; American Psychiatric Association, 2000): dsm.psychiatryonline.org/doi/pdf/10.1176/appi.books.9780890420249.dsm-iv-tr
Space-occupying lesion	Progressive focal neurological symptoms/signs associated with signs/symptoms of increased intracranial pressure, with or without infectious symptoms/signs. Usually slow evolution of symptoms (as opposed to cerebrovascular accident)	Ropper AH, Samuels MA, Klein JP. Adams & Victor's Principles of Neurology. 10th edition. 2014, China. Chapter 30. Disturbances of Cerebrospinal Fluid, Including Hydrocephalus, Pseudotumor Cerebri, and Low-Pressure Syndromes; 617<br>Daroff RB, Fenichel G, Jankovic J, Mazziotta JC. Bradley's Neurology in Clinical Practice. Principles of Diagnosis and Management. 7th edition. 2016, Philadelphia. Chapter 88. Brain Edema and Disorders of Cerebrospinal Fluid Circulation. Rosenberg GA; 1261
Spinal tuberculosis	Neurological symptoms/signs associated with back pain or tenderness, and/or systemic tuberculosis symptoms/signs, and/or paravertebral cold abscess (sometimes with calcification on X-rays), and/or lysis/destruction of	Garg RK, Somvanshi DS. Spinal tuberculosis: A review. *J Spinal Cord Med* 2011; 34(5): 440–454

(continued)

Supplemental Table 3
Continued

Diseases/syndromes	NIDIAG clinical case definition	References/Sources
	intervertebral disk and collapse of adjacent vertebra leading to deformity (kyphosis or gibbus), and/or microbiological evidence of TB infection elsewhere	
Spondylarthropathy, unspecified	Neurological symptoms/signs associated with clinical/radiological evidence of degenerative spondylarthropathy (in the absence of infectious symptoms/signs) and unexplained by "neuropathic syndrome" (see definition of neuropathic syndrome)	NIH – National Institute of Arthritis and Musculoskeletal and Skin Diseases. https:/ ww.ncbi.nlm.nih.gov/pubmedhealth/PMHT0024405/
Temporal arteritis	Headache with/without neurological/visual disturbances and temporal artery abnormality (tenderness/decreased pulse); dramatic clinical improvement with corticosteroids	Buttgereit F et al. Polymyalgia rheumatica and giant cell arteritis: a systematic review. *JAMA* 2016; 315(22): 2442–2458
Tetanus	Acute onset of hypertonia and/or painful muscular contractions (usually of the muscles of the jaw and neck) and generalized muscle spasms without other apparent medical cause (as reported by a health professional)	Tetanus case definitions 2010. Centers for Disease Control and Prevention. https://wwwn.cdc.gov/nndss/.../tetanus/case-definition/2010
Vestibular syndrome	Balance disorder, associated with nausea/vomiting, rotatory vertigo, with or without hearing disturbances (e.g., vestibular neuronitis)	Ropper AH, Samuels MA, Klein JP. Adams & Victor's Principles of Neurology. 10th edition. 2014, China. Chapter 7. Disorders of Stance and Gait; 111<br>Current treatment of vestibular, ocular motor disorders and nystagmus. *Therapeutic Advances in Neurological Disorders* 2009; 2(4): 223–239
Leprosy	Chronic disease characterized by the involvement of mainly skin, peripheral nerves, and the mucosa of the upper airway, ranging from tuberculoid (few hypopigmented and anesthetic skin lesions with sometimes peripheral nerve swelling/thickening) to lepromatous (numerous erythematous or hypopigmented papules/nodules/infiltrative lesions with bilateral and symmetrical distribution; (positive Ziehl staining of skin smear not necessary)	MMWR 1990 Case definitions for Public Health Surveillance: ftp://ftp.cdc.gov/pub/Publications/mmwr/rr/rr3913.pdf<br>WHO Expert Committee on Leprosy: 7th Report, 1998 (http://apps.who.int/iris/handle/10665/42060)
Poliomyelitis, paralytic	Acute onset of asymmetrical flaccid paralysis of one or more limbs with decreased or absent tendon reflexes in the affected limbs, without other apparent cause, and without sensory impairment (as reported by a physician)	MMWR 1990 Case definitions for Public Health Surveillance: ftp://ftp.cdc.gov/pub/Publications/mmwr/rr/rr3913.pdf
Rabies	Acute encephalomyelitis that almost always progresses to coma or death within a highly variable timeframe; notion of animal bite is pivotal	MMWR 1990 Case definitions for Public Health Surveillance: ftp://ftp.cdc.gov/pub/Publications/mmwr/rr/rr3913.pdf

Supplemental Table 4

Metabolic or infectious syndromes with neurological manifestations/complications

Diagnoses	NIDIAG clinical case definition	Source reference
Bacteremia	Isolation of a clinically relevant pathogen in blood culture	–
Hyperglycemia/uncontrolled diabetes	Neurological symptoms related to demonstrated hyperglycemia, with improvement with rehydration and hyperglycemia corrections	National Institute of Diabetes and Digestive and Kidney Diseases (NIDDK) https://www.niddk.nih.gov/health-information/diabetes
Hypertension	Neurological symptoms clearly attributable to high blood pressure (> 150/90 mm of Hg), with clinical improvement with specific anti-hypertension treatment	Hypertension guidelines from the Eighth Joint National Committee (JNC 8) 2014 http://jamanetwork.com/journals/jama/article-abstract/1791497
Hypoglycemia	Neurological symptoms attributable to documented hypoglycemia, with clinical response to glucose challenge	National Institute of Diabetes and Digestive and Kidney Diseases (NIDDK) https://www.niddk.nih.gov/
Lower respiratory tract infection	Neurological symptoms attributable to hypoxemia/sepsis due to (severe) pneumonia (chest X-rays/clinical) or bronchitis (clinical); should include cough, running nose, sore throat, thoracic pain and/or dyspnea	–
Upper respiratory tract infection	Neurological symptoms associated with running nose and/or sore throat and/or fever and/or mouth/ear/sinus pain. Includes also clinical diagnoses of tonsillitis (abnormal/ swollen tonsils), otitis media (abnormal tympanic examination/hypoacousy), sinusitis (sinus pain/tenderness/periorbital cellulitis)	Sinusitis (Rhinosinusitis); https://www.ncbi.nlm.nih.gov/pubmedhealth/PMHT0024567/
Undifferentiated febrile illness (presumptive bacterial)	Neurological manifestations associated with fever/sepsis with no clinical focus of infection, neutrophilic leukocytosis and clear-cut clinical response to antibiotics	–
Undifferentiated febrile illness (presumptive viral)	Neurological manifestations associated with fever with no clinical focus of infection, lymphocytosis and/or leukopenia, and no response to antibiotics or spontaneous resolution	–
